# THE ROLE OF FRAILTY ON THE IMPACT OF SOCIAL RELATIONSHIPS ON HEALTH OUTCOMES: RESULT FROM THE FRÉLE LONGITUDINAL STUDY

**DOI:** 10.1093/geroni/igad104.2094

**Published:** 2023-12-21

**Authors:** Fereshteh Mehrabi, François Béland

**Affiliations:** University of Montreal, Montreal, Quebec, Canada; University of Montreal, Montreal, Quebec, Canada

## Abstract

The link between social relationships and health is well-established as per Berkman and Krishna’s theory. However, the biological mechanisms by which social relationships impact health, such as frailty, remain unknown. This study aimed to examine whether the effects of changes in social relationships on changes in physical, mental, and cognitive health outcomes varied among frail older adults compared to robust peers. Data were from three waves of the FRéLE study among 1643 Canadian community-dwelling older adults aged 65 years and over. We performed latent growth curve models (LGMs) to test our objectives with the assumption of missing not at random. We measured social isolation through social participation, social networks, and support from different social ties (e.g., children, friends). We assessed frailty using the phenotype of frailty. Health outcomes include disability, chronic diseases, depression, and cognitive decline. The results revealed that increasing changes in social participation, social contact with friends, and social support from different social ties were associated with greater changes in cognitive and mental health, but not physical health, among frailer older adults compared to those who were more robust. This longitudinal study suggests that social support has a protective and compensatory role in enhancing mental health among frail older adults, but not among robust peers. Public health policies and interventions should focus on ameliorating social connectedness among physically frail older adults to enhance mental health outcomes. Future research studies could explore other risk factors that impact the relationships between social connectedness and health among older populations.

